# Biomechanical behavior of endocrowns with different axial wall heights: a finite-element study

**DOI:** 10.3389/fdmed.2026.1737491

**Published:** 2026-03-10

**Authors:** Waleed M. S. Al Qahtani, Mohammad Zarbah, Nasser Hussein Shaheen, Ali Barakat, Hend Mohamed Elsayed, Shaimaa F. K. Habib, Lama Ahmed Alqahtani, Ebaa Ibrahim Alagha, Salah A. Yousief, Diaaeldin Farag

**Affiliations:** 1 Prosthetic Dentistry Department, College of Dentistry, King Khalid University, Abha, Saudi Arabia; 2 Restorative and Prosthetic Dental Sciences Department, Dar Al Uloom University, Riyadh, Saudi Arabia; 3 Department of Restorative and Prosthetic Dental Sciences, College of Dentistry, Dar Al Uloom University, Riyadh, Saudi Arabia; 4 Conservative Dentistry Department, Faculty of Dentistry, Cairo University, Cairo, Egypt; 5 College of Dentistry Graduate, Dar Aluloom University, Meras Medical Company Clinic, Riyadh, Saudi Arabia; 6 Dental Medicine and Surgery Department, Faculty of Dentistry, Vision Medical College, Jeddah, Saudi Arabia; 7 Department of Crown and Bridge, Faculty of Oral and Dental Medicine, Al Azhar University, Cairo, Egypt; 8 Fixed Prosthodontic Department, Faculty of Dentistry, Suez Canal University, Ismailia, Egypt; 9 Restorative Dental Sciences Department, College of Dentistry, Gulf Medical University, Ajman, United Arab Emirates

**Keywords:** butt joint, CAD-CAM, crowns, digital analysis, endocrown, finite element, FPD, shoulder finish line

## Abstract

**Introduction:**

The aim of this study was to compare endocrown restorations of mandibular first molars using four distinct preparation designs with different residual tooth heights by employing finite-element analysis. A butt joint marginal design was compared with three shoulder finish line preparations.

**Materials and methods:**

Four three-dimensional finite-element models were developed to represent different endocrown configurations. Model 1 simulated a butt joint margin design, whereas Models 2, 3, and 4 represented shoulder finish line designs with remaining tooth structure heights of 1, 2, and 3 mm, respectively. Two restorative materials, zirconia and IPS e.max® computer-aided design, were evaluated. The geometry of a healthy mandibular first molar was obtained from computed tomography data and reconstructed using SolidWorks®. Cortical and cancellous bone were modeled as coaxial cylinders. Two loading conditions were applied, i.e., a 400 N vertical load and a 200 N oblique load at 45°, both of which were directed to the buccal cusp tips and central fossa**.**

**Results:**

Under vertical loading, the butt joint design exhibited the lowest von Mises stress values, whereas the shoulder finish line designs showed progressively higher stresses with increasing axial wall height. The butt joint design also reduced stresses in the cortical bone by up to 15% compared with the shoulder finish line designs. The choice of restorative material had minimal influence on stress distribution; however, zirconia crowns demonstrated slightly higher stress values, suggesting greater material durability**.**

**Conclusion:**

Endocrowns with a butt joint margin demonstrated superior biomechanical behavior by minimizing stress transfer to the underlying tooth and supporting bone. The influence of the endocrown’s design decreased toward the alveolar bone, while the restorative material only exerted a minor effect on the stress distribution.

## 1 Introduction

Restoration principles following endodontic treatment must respect the remaining tooth structure and aim to achieve optimal esthetics, proper function, and accurate anatomical form. The primary treatment goals include minimizing tooth preparation and preserving healthy dental tissues. These principles enhance the biomechanical performance of the restored tooth complex and increase the available bonding surface for adhesive restorative procedures.

All-ceramic restorations are fabricated from advanced ceramic materials and do not require a metal substructure for support; therefore, they offer excellent biocompatibility and superior esthetic outcomes. Based on their compositional architecture and fabrication process, all-ceramic systems can be broadly classified into two structural categories (1, 2).

The first category includes monolithic all-ceramic restorations, which consist of a single, homogeneous ceramic structure throughout the entire restoration. This monolithic approach utilizes the same ceramic material from the internal surface to the external contour, resulting in a seamless restoration without layered interfaces or compositional differences.

The second category comprises bilayer all-ceramic restorations, which incorporate a high-strength ceramic framework or substructure (i.e., core) that is subsequently veneered with an esthetic ceramic layer. This bilayered configuration combines the mechanical strength of the core material with the superior optical and esthetic properties of veneering ceramics, thereby optimizing both functional performance and esthetic outcomes (2).

Within this classification, contemporary computer-aided design and computer-aided manufacturing (CAD/CAM) endocrown restorations are predominantly fabricated using monolithic ceramic materials and rely on adhesive bonding for retention and stress distribution. Lithium disilicate ceramics are characterized by a relatively lower elastic modulus and strong bonding potential following surface conditioning, making their biomechanical behavior highly dependent on the integrity of the adhesive interface. In contrast, zirconia ceramics exhibit a significantly higher elastic modulus and superior fracture resistance, resulting in greater structural stiffness and reduced reliance on adhesive bonding but with a higher potential for stress transfer to the tooth–restoration interface. These material-specific characteristics suggest that lithium disilicate and zirconia endocrowns may exhibit different biomechanical responses under functional loading.

Despite advances in restorative materials, one of the most significant challenges faced by dental practitioners remains the restoration of endodontically treated teeth with extensive loss of tooth structure. This challenge arises from the structural alterations associated with root canal treatment, which lead to reduced tooth stiffness and diminished fracture resistance. Consequently, these compromised teeth exhibit a substantially higher risk of biomechanical failure compared with healthy teeth (3).

Ceramics are inert materials characterized by excellent biocompatibility and a high degree of intraoral stability; therefore, they can be safely used in the oral cavity (2). Marginal adaptation refers to the accuracy of the interface between the restoration and the prepared tooth structure. The clinical outcome of endocrown restorations is strongly influenced by both marginal and internal adaptation. Defective adaptation may result in bacterial microleakage, which, over time, can lead to degradation of the luting cement. This process may subsequently cause endodontic and periodontal inflammation and ultimately compromise the longevity of the prosthesis (4).

However, investigations examining the influence of different restorative materials on restoration fit have reported contradictory findings. Several studies have concluded that ceramic restorations demonstrate superior marginal adaptation and internal fit compared with composite resin restorations, whereas other authors have reported conflicting results (5).

In this context, CAD/CAM systems are a fundamental component of contemporary digital dentistry workflows and significantly improve efficiency and precision in restorative procedures. This streamlined chairside workflow, allowing for same-day restoration from digital impression to final cementation, combined with superior esthetic outcomes and clinically acceptable biomechanical properties, has established endocrown restorations as a widely accepted treatment option for restoring extensively damaged posterior teeth following endodontic treatment (4, 5).

Longitudinal clinical investigations have reported high survival and success rates for CAD/CAM-fabricated endocrowns, although these outcomes are study-specific and influenced by follow-up duration and restorative material. Belleflamme et al. (6), in a retrospective study with a follow-up of up to 10 years, reported favorable long-term performance for endocrowns used to restore severely damaged posterior teeth without post–core systems. Similarly, Zou et al. (7) reported a 100% clinical success rate for CAD/CAM-fabricated monolithic zirconia endocrowns in molars with extensive coronal loss over a 3-year observation period. More recently, Do et al. (8) demonstrated a favorable short-term clinical survival rate (96.4%) for lithium disilicate CAD/CAM endocrowns over a 2-year follow-up period. Despite these encouraging results, recurrent caries and loss of retention have been identified as the most common modes of failure, often associated with degradation of the cement interface and inadequate marginal or internal adaptation. Therefore, a comprehensive understanding of the biomechanical and design-related factors influencing endocrown performance remains essential for predictable long-term outcomes (5).

Polymerization shrinkage is a major cause of failure in resin-based dental restorations (3). The success of ceramic restorations depends on several critical factors, including material selection, endocrown design, and the characteristics of the adhesive interface (1).

Accordingly, the aim of this study was to compare endocrown restorations of mandibular first molars using four distinct preparation designs with different residual tooth heights through finite-element analysis (FEA), comparing a butt joint marginal design with three shoulder finish line preparations. In addition, the biomechanical performance of two ceramic endocrown systems, IPS e.max® CAD (Ivoclar Vivadent, Schaan, Liechtenstein) lithium disilicate and zirconia, was evaluated, as these materials represent the most clinically established CAD/CAM restorative ceramics for posterior endocrown fabrication.

## 2 Materials and methods

### 2.1 Finite-element model development

**Figure 1:**
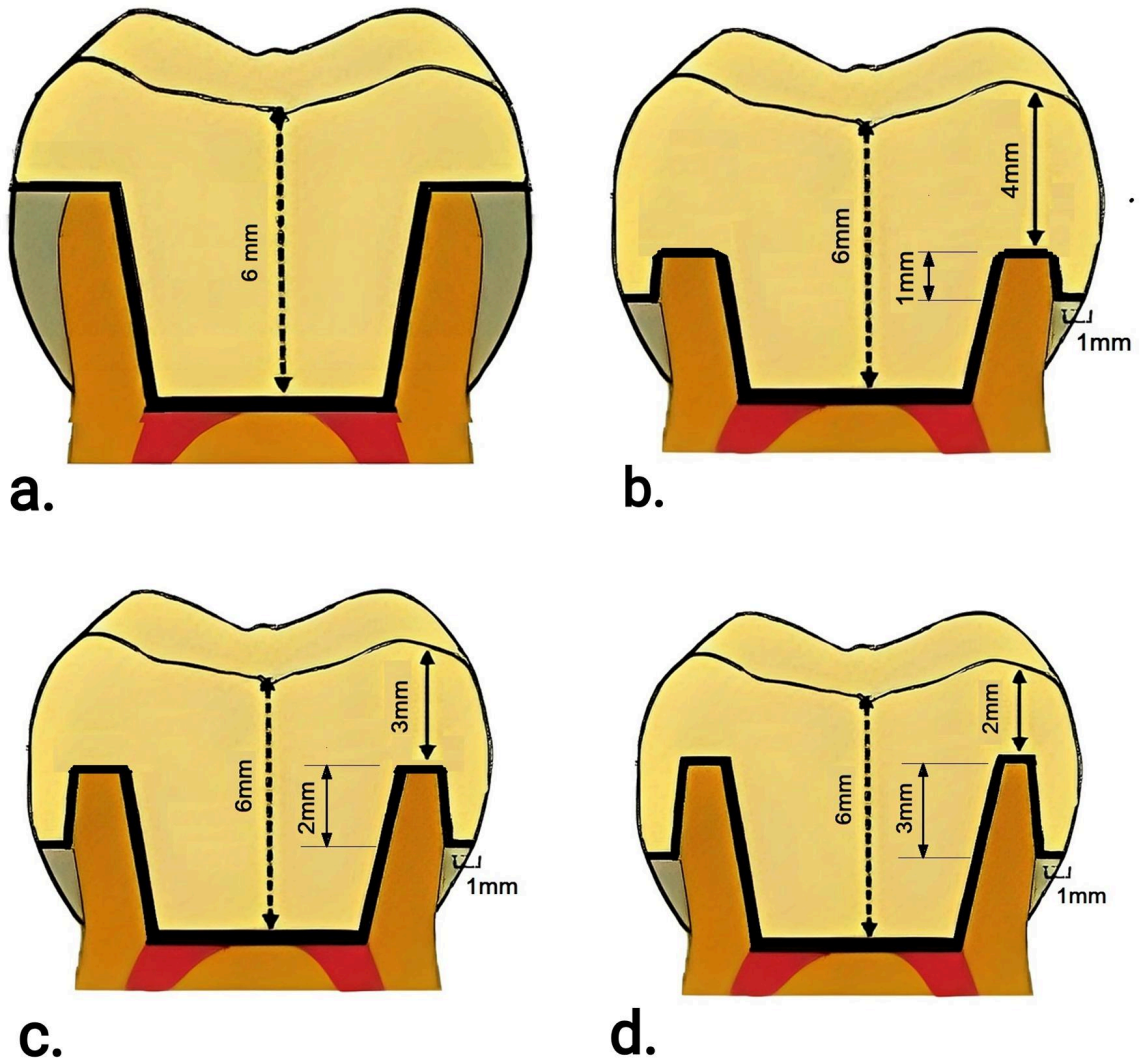
Schematic drawing for the four studied cases: **(a)** Model #1: butt joint; **(b)** Model #2: shoulder finish line with a remaining tooth height of 1 mm; **(c)** Model #3: shoulder finish line with a remaining tooth height of 2 mm; and **(d)** Model #4: shoulder finish line with a remaining tooth height of 3 mm.

**Figure 2:**
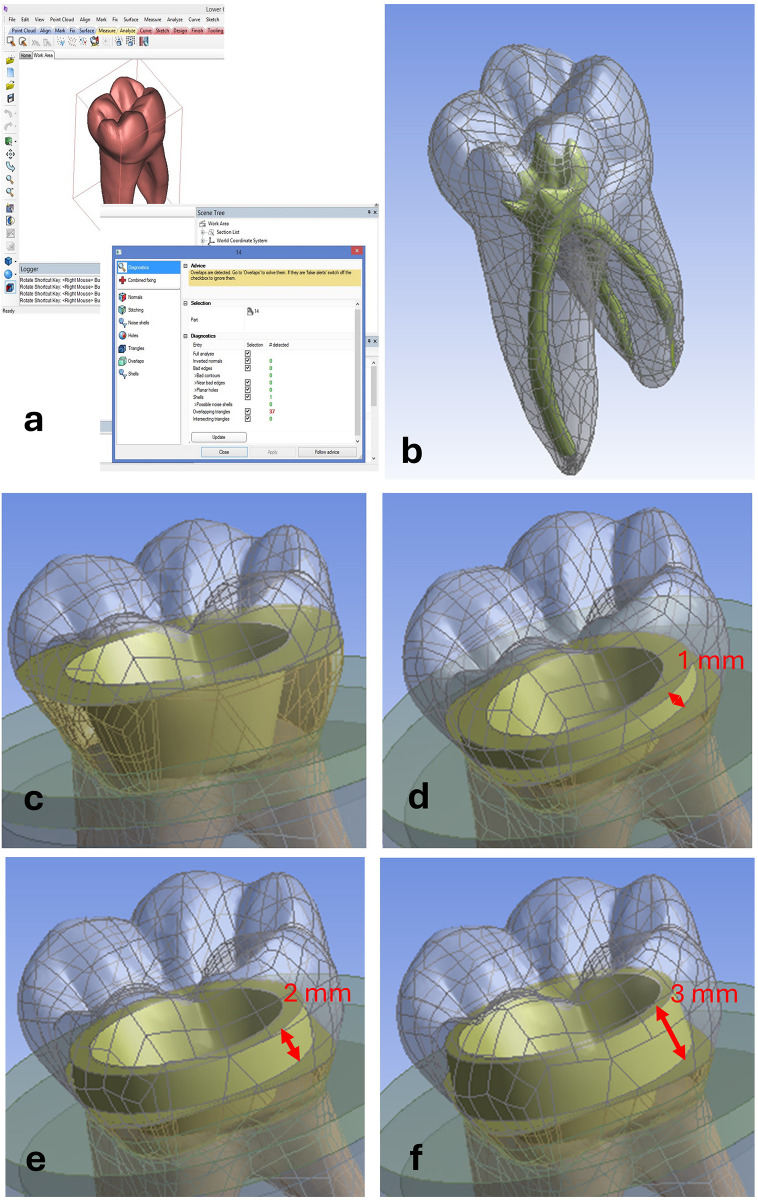
A healthy tooth and the four created models (designs): **(a)** STL file corrections of the healthy tooth on 3-matic and **(b)** its solid model in ANSYS (gray: tooth structure, dark green: pulp chamber/root canal space); **(c)** Model #1: butt joint margin design; **(d)** Model #2: shoulder finish line with a remaining tooth height of 1 mm; **(e)** Model #3: shoulder finish line with a remaining tooth height of 2 mm; and **(f)** Model #4: shoulder finish line with a remaining tooth height of 3 mm. The red arrows indicate the remaining axial tooth heights.

Four distinct three-dimensional finite element (FE) models were constructed to simulate different endocrown restoration configurations and evaluate their biomechanical behavior. Model 1 represented a butt joint marginal design, whereas Models 2, 3, and 4 represented shoulder finish line preparations with remaining coronal tooth heights of 1, 2, and 3 mm, respectively (Figures 1, 2).

### 2.2 Model generation and geometric reconstruction

A clinically healthy mandibular first molar was selected as the reference tooth for three-dimensional model generation. The tooth’s geometry was acquired using high-resolution computed tomography imaging and subsequently converted into standard triangle language (STL) format for digital processing (Figure 2A). The STL datasets were processed using 3-matic (version 15.01, Materialise NV, Leuven, Belgium) to correct mesh errors, eliminate redundant data points, and optimize both the external and internal tooth morphologies. Following geometric refinement, the final solid model was exported to SolidWorks® (version 2014, Dassault Systèmes, Vélizy-Villacoublay, France) for further design modification and model preparation. The pulp chamber extension geometry, including internal wall angulation, was standardized across all models to isolate the effect of axial wall height on the stress distribution and ensure consistent geometric conditions for the comparative analysis.

### 2.3 Anatomical structure integration

The finite-element model comprised several anatomical components, including enamel; dentin; a cement interface layer (20 μm in thickness); a periodontal ligament (PDL) modeled as a uniform 0.3 mm layer surrounding the tooth root, consistent with previously validated finite-element studies reporting physiological PDL thickness values ranging between 0.2 and 0.4 mm (9); and supporting alveolar bone structures. The cortical and cancellous bones were modeled as concentric cylindrical structures with standardized dimensions (outer diameter: 18 mm, inner diameter: 14 mm, total height: 26 mm). This simplified geometry was used purely as a computational assumption to standardize the boundary conditions across all the finite-element models and does not reflect a density-based anatomical or biological classification of the alveolar bone.

### 2.4 Model assembly and analysis preparation

All the anatomical and restorative components were systematically assembled into unified geometric models and exported as standard for the exchange of product data (STEP) files for subsequent biomechanical analysis using ANSYS Workbench (version 16.0, ANSYS Inc., Canonsburg, PA, USA) (Figures 2B–E).

### 2.5 Material properties

All the materials included in this study were assumed to be homogeneous, isotropic, and linearly elastic, in accordance with previous FEA studies (10, 11). This assumption was used to standardize the comparisons between the different endocrown designs under identical conditions (Table 1).

**Table 1:** Material properties of the materials used in the finite-element model(s).

Material	Young's modules (GPa)	Poisson's ratio
Zirconia crown	210	0.35
IPS e.max® CAD crown	97.5	0.24
Resin cement	4.04	0.30
Gutta-percha	0.00069	0.45
Enamel	84.1	0.31
Dentin	18.6	0.31
Mucosa	0.01	0.40
Cortical bone	13.7	0.30
Cancellous bone	1.37	0.30

The elastic modulus (E) and Poisson's ratio (*ν*) of each material were assigned based on values reported in the literature (Table 1). The following two crown materials were evaluated: zirconia (*E* = 210 GPa, *ν* = 0.35) and IPS e.max® CAD (*E* = 97.5 GPa, *ν* = 0.24). The remaining components, namely, dentin, enamel, cement, cortical bone, cancellous bone, mucosa, and gutta-percha, were modeled using their respective mechanical properties.

### 2.6 Finite-element meshing and boundary conditions

**Figure 3:**
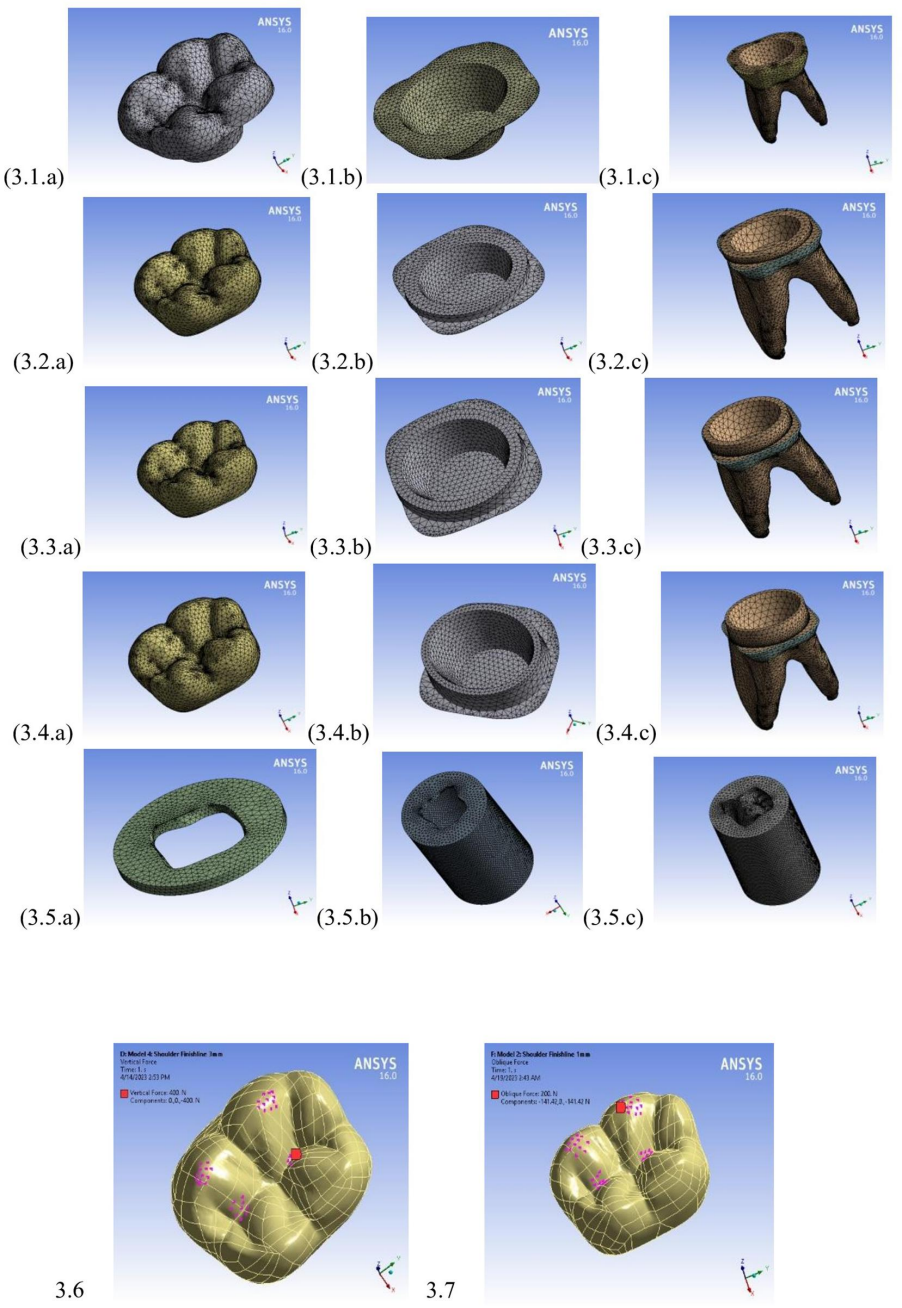
Meshed models’ components: (3.1) Model #1, (3.2) Model #2, (3.3) Model #3, (3.4) Model #4, and (3.5) common components. The applied loading conditions are illustrated as (3.6) vertical load applied along the long axis and (3.7) 45° oblique load applied to the occlusal surface.

Each component of the assembled models was meshed using quadratic tetrahedral elements (SOLID187). A mesh convergence test was performed by progressively refining the mesh density until the variations in the maximum von Mises stress within the cortical bone were less than ±3%, thereby ensuring numerical stability and accuracy. The resulting numbers of nodes and elements are summarized in Table 2, and representative views of the meshed components are shown in Figure 3.

**Table 2:** Number of nodes and elements in all the meshed components.

Volume	Model 1: endocrown with butt joint design		Model 2: endocrown with a remaining tooth height of 1 mm		Model 3: endocrown with a remaining tooth height of 2 mm		Model 4: endocrown with a remaining tooth height of 3 mm	
	Number of nodes	Number of elements	Number of nodes	Number of elements	Number of nodes	Number of elements	Number of nodes	Number of elements
Crown	234,854	170,608	257,656	187,130	257,362	186,826	253,780	184,013
Cement	21,459	10,393	16,486	7,977	18,033	8,749	20,507	9,977
Enamel	17,461	9,875	9,343	5,133	9,343	5,133	9,343	5,133
Dentin	138,959	139,371	236,033	167,871	245,137	174,238	236,197	167,235
Mucosa	30,053	19,573	30,053	19,573	30,053	19,573	30,053	19,573
Cortical bone	108,796	64,472	108,796	64,472	108,796	64,472	108,796	64,472
Cancellous bone	365,419	258,963	365,640	257,636	363,640	257,636	363,640	257,636

Boundary conditions were applied by fixing the inferior surface of the cortical bone cylinder in all directions (*x*-, *y*-, and *z*-axes) to simulate anatomical support. The cement layer was assumed to be perfectly bonded to both the crown and dentin interfaces to represent ideal adhesive conditions.

### 2.7 Loading conditions and validation

The following two static loading conditions were applied to each model:

1. vertical loading: a 400 N load applied perpendicular to the occlusal surface, and
2. oblique loading**:** a 200 N load applied at a 45° angle to the buccal cusp tips and central fossa to simulate masticatory forces.

Both loading conditions were applied over small circular contact areas to replicate realistic occlusal stress distribution. These load magnitudes represent the average functional forces encountered in the posterior dentition during mastication. A linear static analysis was performed using a workstation (HP Z820; Dual Intel Xeon E5-2670 v2 processors, 2.5 GHz, 64 GB RAM) and a commercial multipurpose finite-element software package (ANSYS, version 16.0) (Figure 3). The results obtained from these models were verified through comparison with previously published studies (10, 12).

## 3 Results

**Figure 4:**
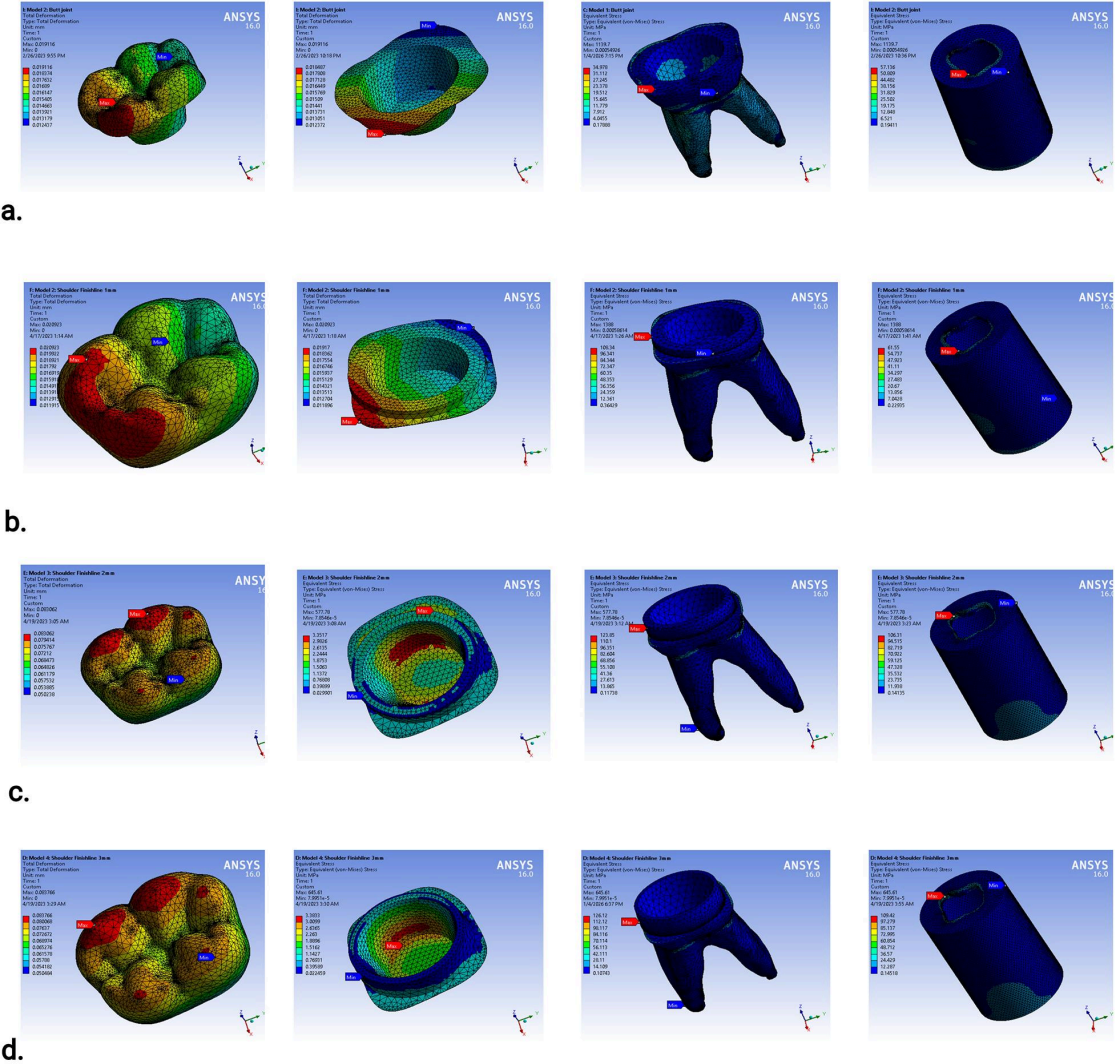
Sample of analyses results as screen shots from ANSYS: **(a)** butt joint margin model with IPS e.max® CAD endocrown; **(b)** shoulder finish line with 1 mm axial wall height with IPS e.max® CAD endocrown where both were subjected to vertical loading; **(c)** shoulder finish line with 2 mm axial wall height with zirconia endocrown; and **(d)** shoulder finish line with 3 mm axial wall height with zirconia endocrown where both were subjected to oblique loading.

**Figure 5:**
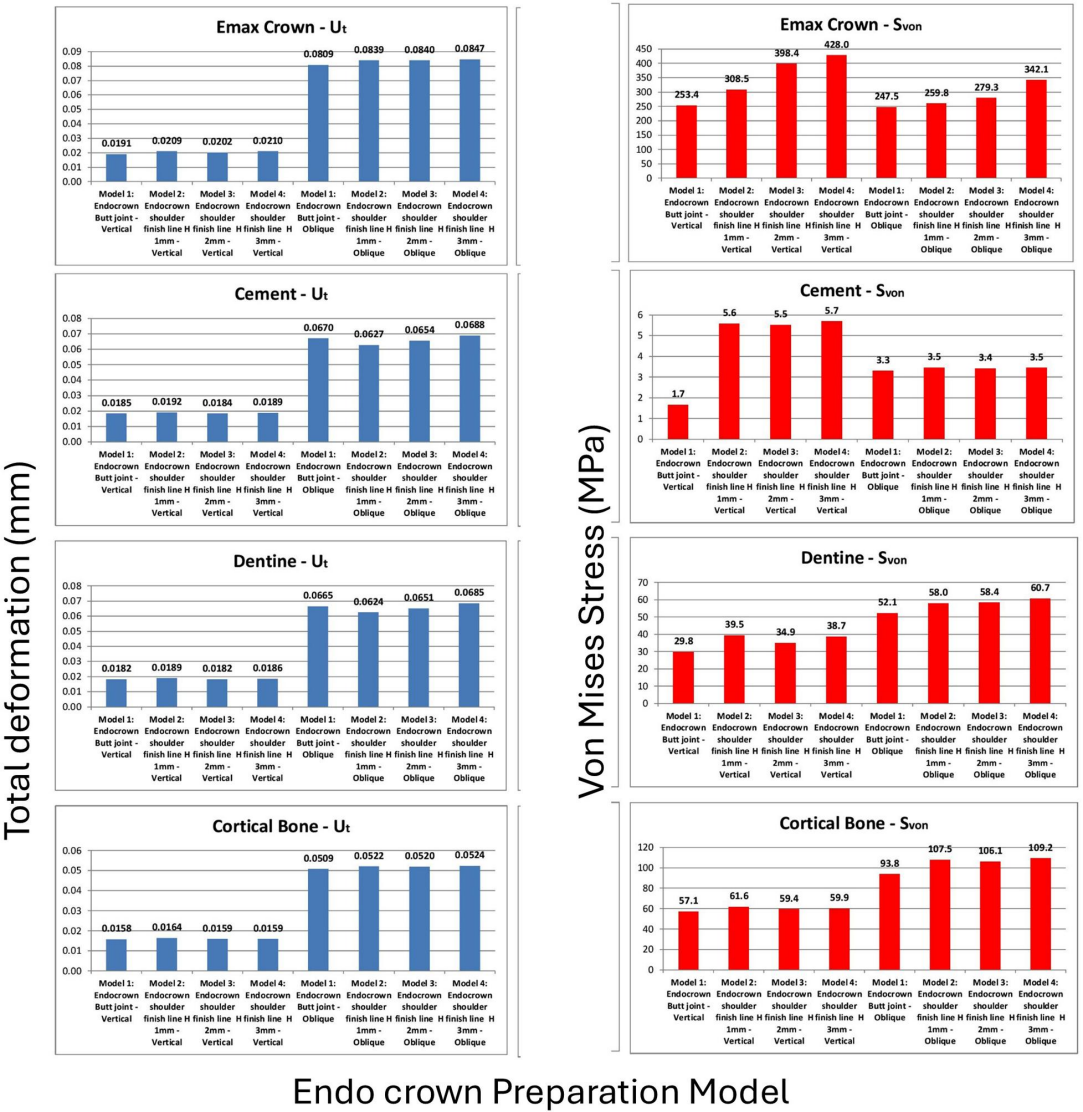
Comparisons of extreme values of total deformation (mm) and von Mises stress (MPa) with IPS e.max® CAD crowns: crown, cement layer, dentine or remaining tooth, and cortical bone.

**Figure 6:**
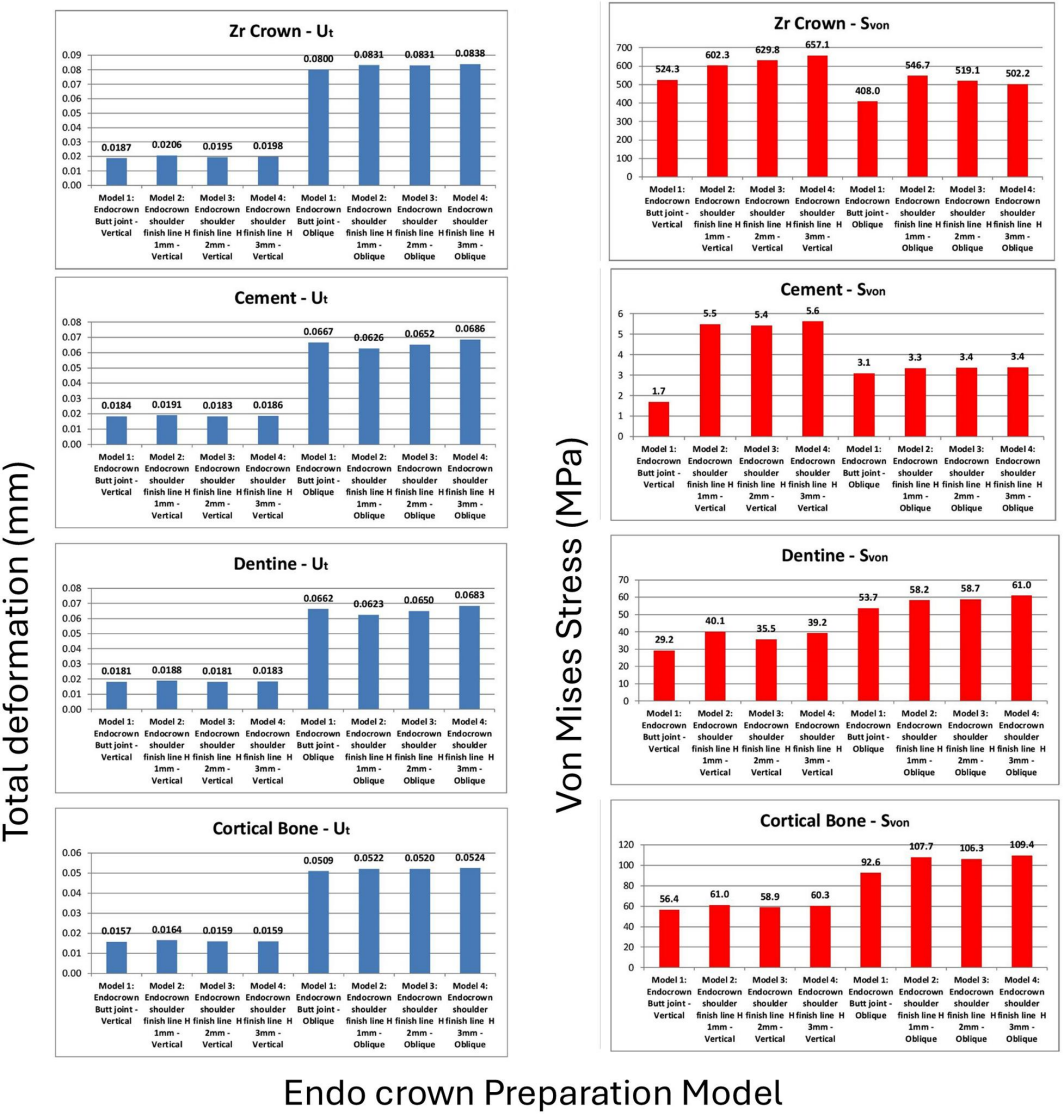
Comparisons of extreme values of total deformation (mm) and von Mises stress (MPa) with zirconia crowns: crown, cement layer, dentine or remaining tooth, and cortical bone.

A total of 16 finite-element analyses were performed, corresponding to two loading conditions applied to the two endocrown materials across the four preparation designs. Overall, the results were within comparable ranges, with no unexpected or unrealistic variations among the tested models. Due to the large volume of graphical output, representative stress and deformation distributions are presented in Figure 4. Comparative analyses of total deformation and von Mises stress between the different designs and loading conditions are illustrated in Figure 5 for IPS e.max® CAD crowns and Figure 6 for zirconia crowns.

### 3.1 IPS e.max® CAD endocrowns

For the IPS e.max® CAD endocrowns (Figure 5), the differences in crown total deformation among the four models under each loading condition did not exceed 2 µm. In contrast, the von Mises stress values within the crown increased progressively with increasing axial wall height, with clear numerical differences among the evaluated designs. The butt joint design exhibited the lowest von Mises stress values, followed by the shoulder finish line design with the 1 mm axial wall height, which demonstrated stress increases of approximately 20% under vertical loading and 5% under oblique loading. The shoulder finish line designs with greater axial wall heights exhibited higher stress concentrations within the crown and at the tooth–restoration interface compared with the butt joint configuration.

The cement layer did not exhibit critical stress concentrations in any model; under vertical loading, the von Mises stress values ranged from approximately 1.7 MPa in the butt joint design to 5–6 MPa in the shoulder finish line designs, while under oblique loading, the values were approximately 3–4 MPa across all models.

The remaining tooth structure showed minimal differences in total deformation and did not exceed 8% in any of the four designs under all loading conditions. The von Mises stress values within the dentin were also comparable among the models, with the butt joint design consistently demonstrating the lowest stress values. Interestingly, increasing the axial wall height did not proportionally increase dentinal stress; Model 3 (2 mm axial wall height) exhibited slightly lower stress values under vertical loading and stress values comparable to Models 2 and 4 under oblique loading, with only minor numerical differences among these designs. Importantly, all the dentinal stress values remained within physiological limits.

The stress and deformation trends observed in the cortical bone followed a pattern similar to that of the dentin. Under oblique loading, the von Mises stress values approached critical limits; however, the butt joint design consistently demonstrated up to 15% lower stress values compared with the shoulder finish line designs. This finding indicates that the biomechanical advantage of the butt joint margin extends from the restoration to the supporting bone.

### 3.2 Zirconia endocrowns

For zirconia endocrowns (Figure 6), there were no significant differences in total crown deformation between the four preparation designs. However, the von Mises stress distribution within the crown exhibited two distinct behaviors depending on the loading condition. Under vertical loading, increasing axial wall height resulted in higher crown stress values, whereas under oblique loading, increasing axial wall height led to decreasing crown stress.

The cement layer, remaining tooth structure, and supporting bone demonstrated negligible sensitivity to the endocrown material. The differences in total deformation and von Mises stress between the zirconia and IPS e.max® CAD models were minimal. Therefore, no additional comparative analysis of the supporting structures under the zirconia crowns is presented.

## 4 Discussions

This FEA investigated the biomechanical behavior of mandibular first molars restored with endocrowns featuring different margin designs and remaining coronal tooth heights. The results demonstrated that the butt joint margin configuration produced the most favorable stress distribution, exhibiting lower von Mises stress values in both the restorative components and supporting structures compared with the shoulder finish line preparations. The shoulder finish line designs with greater axial wall heights exhibited higher stress concentrations within the crown and at the tooth–restoration interface compared with the butt joint configuration. These findings indicate less favorable biomechanical behavior under the simulated loading conditions; however, they should not be interpreted as direct predictors of clinical service life, which is influenced by multiple biological, mechanical, and patient-related factors.

The observed reduction in stress within the butt joint configuration can be attributed to its extensive, flat contact surface, which effectively disperses occlusal forces across the adhesive interface. This design promotes a gradual transfer of stress from the restoration to the tooth structure, allowing forces to be distributed over a larger surface area. Consequently, stress concentration at the cervical region is minimized, and compressive forces are directed along the longitudinal axis of the tooth. These findings are consistent with those reported by Lin et al. (13) and Cheng et al. (14).

In comparison, shoulder finish line preparations create a more abrupt transition between the restorative material and the tooth structure, leading to increased tensile stress concentration at the cervical margin. Furthermore, increasing axial wall height exacerbates these stresses due to a cantilever-like effect, which generates higher bending moments transmitted to the dentin and cortical bone. Beyond axial wall height, the geometry of the pulp chamber extension, particularly internal wall angulation, also plays a critical role in the biomechanical behavior of endocrown restorations. Steeper or sharper extension angles are associated with increased tensile stress concentrations at internal line angles, which is of particular concern for lithium disilicate ceramics due to their brittle nature and sensitivity to tensile loading. In contrast, more divergent or smoothly rounded pulp chamber extensions promote a more favorable stress distribution by reducing the stress intensification within the ceramic bulk and at the adhesive interface (15). In addition, the presence of stress-absorbing filling materials, such as resin composite bases replacing missing dentin, may further modulate the stress transfer and attenuate the peak stresses transmitted to lithium disilicate endocrowns, thereby potentially enhancing their fracture resistance and adhesive stability (16).

Furthermore, the butt joint design offers distinct advantages by conserving more of the coronal tooth structure. Preservation of coronal dentin is critical for maintaining the structural integrity of endodontically treated teeth. In contrast, shoulder finish line preparations require the removal of a larger volume of dental tissue, which may compromise adjacent tooth structures and create regions more susceptible to stress concentration (2). In this study, the butt joint configurations reduced cortical bone stress by up to 15%, further supporting their biomechanical advantage.

Biomechanically, shoulder finish line preparations may adversely affect stress distribution under both vertical and oblique loading conditions, despite their traditional use to improve marginal adaptation. The butt joint design provides a more stable and uniform interface that more effectively resists compressive and shear forces due to its orientation parallel to the occlusal plane. These findings corroborate previous reports emphasizing the importance of enamel preservation and conservative cavity preparation in maintaining favorable stress behavior (17, 18).

The comparison between zirconia and lithium disilicate (IPS e.max® CAD) endocrowns revealed minimal differences in stress distribution patterns. Although zirconia exhibited slightly higher stress values within the crown, attributable to its higher elastic modulus, the stress levels in the underlying tooth structure and supporting bone were comparable between the materials. This observation suggests that once a critical stiffness threshold is achieved, the influence of the restorative material on stress transfer becomes limited (5, 19). Consequently, material selection for endocrowns should be guided primarily by esthetic demands and clinical considerations rather than biomechanical differences alone.

These findings reinforce the concept that conservative endocrown preparations with butt joint margins are not only minimally invasive but also biomechanically favorable. The flat occlusal interface increases the adhesive bonding surface, reduces polymerization stress within the cement layer, and minimizes the risk of debonding. Such characteristics are particularly relevant in molar regions, where functional occlusal forces may reach 800–900 N (18). Notably, stress variations among the tested designs diminished progressively from the crown–cement interface toward the alveolar bone, indicating that the design-related effects are primarily localized within the restoration and coronal tooth structure. This observation aligns with clinical reports suggesting that most endocrown failures originate at the adhesive interface rather than within the root or supporting bone (20).

Our results also underscore the importance of appropriate case selection. Endocrowns are most suitable for molars with adequate enamel margins and a sufficient pulp chamber depth (>3 mm) to ensure optimal bonding conditions. In cases involving high lateral or parafunctional loading, conventional full-coverage crowns supported by posts may still offer more predictable outcomes. Nevertheless, the minimally invasive nature of endocrowns, preserving pulp chamber walls and avoiding post placement, remains a significant advantage in reducing the risk of root fracture.

Previous finite element and *in vitro* studies have consistently demonstrated that butt joint and chamfer-type designs reduce stress concentrations at the tooth–restoration interface compared with shoulder preparations (13, 12).

This study extends previous findings by quantifying the effect of incremental increases in axial wall height on the biomechanical behavior of endocrown restorations. Specifically, the 2 mm shoulder design exhibited stress values comparable to the butt joint configuration, whereas the 3 mm axial wall height resulted in the highest stress concentrations, suggesting that excessive wall height without adequate enamel support may be counterproductive. From a biomechanical perspective, an intermediate axial wall height appears to provide a favorable balance between geometric retention and efficient stress transfer. Under oblique loading, which generates higher bending moments and shear stresses at the cervical region, the 2 mm axial wall height facilitated a more uniform distribution of stresses along the tooth–restoration interface. In contrast, lower axial walls offered limited lateral support, while higher axial walls may have acted as a cantilever, amplifying bending stresses under non-axial forces. These findings are consistent with the conclusions of Huang et al. (3), who emphasized the importance of enamel continuity for optimal stress transfer and adhesion.

Overall, the findings of this study are consistent with previous investigations while providing novel insights into endocrown margin design. Lin et al. reported more favorable stress distributions with conservative preparation designs in endodontically treated molars but did not specifically compare margin configurations within endocrown restorations (13). Cheng et al. demonstrated that both material properties and geometric parameters influence endocrown performance, although our results suggest that margin configuration may play a more dominant role than material selection (14). Similarly, Taha et al. showed that margin design significantly affects fracture resistance and failure modes, supporting the superior performance of conservative designs (18). Unlike these studies, which focused primarily on polymer-infiltrated ceramics, this investigation evaluated zirconia and lithium disilicate materials.

Based on these findings, we propose a clinical decision-making framework for endocrown margin design. For teeth with sufficient coronal structure (≥2 mm), butt joint preparations should be considered the preferred option due to their conservative nature and superior biomechanical behavior. Shoulder finish line preparations should be reserved for cases with specific esthetic demands or anatomical constraints. Given the minimal influence of material type on stress distribution, selection between zirconia and lithium disilicate should be based primarily on esthetic requirements and clinician preference.

## 5 Limitations and future directions

This finite-element analysis includes several inherent limitations that should be considered when interpreting the results. First, all the materials in the models were assumed to be homogeneous, isotropic, and linearly elastic; however, natural dental tissues and ceramics exhibit anisotropic and viscoelastic behaviors. This simplification may lead to an underestimation of localized stress concentrations, particularly at internal angles and adhesive interfaces. Second, static loading conditions were used, whereas true masticatory forces are cyclic, variable in magnitude, and multidirectional. As a result, fatigue-related stress accumulation and long-term degradation may not be fully represented, potentially underestimating areas susceptible to failure under functional loading. Third, perfect bonding was assumed at all cement–restoration and cement–dentin interfaces. In clinical reality, microgaps, adhesive degradation, and partial debonding may occur, which could increase the interfacial stresses beyond those predicted in this study. Finally, the cortical and cancellous bones were represented using an idealized concentric cylindrical geometry based on a computational assumption to standardize the boundary conditions rather than reflect true anatomical variability or a density-based classification, which may influence the accuracy of the stress transmission patterns within the supporting structures.

Accordingly, future research should incorporate anisotropic material properties, cyclic loading protocols, and more anatomically realistic boundary conditions, in addition to *in vitro* mechanical testing and well-designed clinical studies, to validate the present finite-element findings and better assess the long-term clinical performance of different endocrown designs and materials under realistic oral conditions.

## 6 Conclusions

Based on the findings of this finite-element analysis, the following conclusions can be drawn:

1. Endocrowns with a butt joint margin design demonstrated superior biomechanical performance compared with shoulder finish line preparations.
2. Among shoulder finish line designs, a moderate axial wall height (2 mm) exhibited lower stress concentrations, whereas a 1 mm axial wall height showed slightly higher stresses, and a 3 mm axial wall height resulted in the highest stress concentrations.
3. The choice of endocrown material had a negligible or minor influence on the stress distribution within the underlying tooth structure and supporting bone.

The endocrown technique is a simple, time-efficient, and minimally invasive restorative approach. As the root canals are not involved in the procedure, trauma to the remaining tooth structure is minimized. Overall, endocrowns are a promising restorative option for endodontically treated multirooted teeth.

## Data Availability

The original contributions presented in the study are included in the article/Supplementary Material, further inquiries can be directed to the corresponding author/s.
